# Mucin

**Published:** 1905-07-08

**Authors:** 


### HOSPITAL CLINICS.

### MUCIN.

MR. STUART-LOW has published, in the shape of a small book,[1] a lecture delivered before medical graduates at the Polyclinic College upon the subject of mucous membranes, and what he considers their chief secretion, mucin. The claims of mucin to this proud position have undoubtedly escaped acceptance from physiologists, and we fear the lecture under notice will hardly effect any pronounced alteration in the status of this obscure body. The largest mucous surface in the body is the alimentary canal, and to say that mucin is its chief secretion places a severe tax upon the credulity of those brought up in the common teachings of physiology. This doubt must remain until some better evidence than that afforded by this publication shall arrive to disperse it.

A considerable portion of the book is occupied with an anatomical review of mucous membranes in general, though, naturally enough, the pen of a devotee of the nose and throat dwells upon the area of his special intimacy with a fondness which may seem disproportionate to the general observer. None the less we are grateful for his bias, for this section of the subject contains more facts than the rest of the book together, but the author's inferences fail to command our confidence. For instance, in the section on Orthomyxia, which signifies a healthy condition of the mucous membranes, Mr. Stuart-Low observes, "I have lately been examining a number of very old people, mostly old men. I have found them all wonderfully orthomyxiatous, having healthy naso-pharyngeal mucous membranes, good digestion and regular bowel action. I am inclined to believe, therefore, that such is conducive to healthy old age. I certainly have not yet seen one healthy old man who was not orthomyxiatous, so that one is warranted in reckoning this as a condition favouring longevity." Strange, that old men who have proved the robustness of their constitutions should be found with healthy mucous membranes! Or has the author neglected the difference between *post* and *propter*?

In the section on Pathology we are told how the author became impressed by the frequency with which patients suffering from gastric irritability, painful digestion, and hæmatemesis, suffered simultaneously from either dry or atrophic rhinitis or pharyngitis, and conversely, that almost all those suffering from dryness of the nose and throat complained also of long-standing and intractable gastralgia and constipation of the bowels. We feel sure that this cannot be the general experience. No type of case better exemplifies the condition of painful digestion and hæmatemesis than the chlorotic girl who forms so noticeable a feature in the casualty and out-patient departments of a general hospital. We do not pretend to have examined the noses of such patients with anything approaching regularity, but the tongue in this condition is, in our experience, almost invariably pale, moist and flabby, and does not suggest a deficiency of mucus in the salivary contents at all events.

In discussing the pathological conditions of the mucous secretion Mr. Stuart-Low has adopted a nomenclature suggested by Dr. Overend Walker. Orthomyxia indicates, as we have said, a healthy state of the secretion; hypermyxia an excessive, and hypomyxia a deficient activity of the membrane in regard to its output of mucus. The type of hypermyxia is set by hypertrophic rhinitis, though the condition may be local or general. Of the latter we are told nothing; but a section is devoted to the treatment of hypertrophic rhinitis, and the following lotion is recommended for the removal of accumulated secretions :—

| | |
|---|---|
| Sodii chlor. ... ... ... ... | gr. 2 |
| Sodi bibor. ... ... ... ... | gr. $1\frac{1}{2}$ |
| Sodii benzoat ... ... ... ... | gr. $\frac{1}{4}$ |
| Menthol crys. ... ... ... ... | gr. $\frac{1}{200}$ |
| Cocain. hydrochlor. ... ... ... | gr. $\frac{1}{20}$ |
| Aquæ ad ... ... ... ... ... | ℥ j |

This lotion should be used warm to irrigate the nasal passages occasionally.

The type of hypomyxia is rhinitis sicca. In this, as in all cases where the supply of mucus appears defective, it is advised that an attempt should be made to supply the lacking material by the exhibition of mucin, both internally and as an application to the affected surfaces. "The topical application of mucin is soothing and emollient; it is hygroscopic and therefore moistening, and serves to remove dryness and discomfort. When the local condition is well marked the solution of soloid mucin compound is used, . . . but in mild cases a solution of the tabloid mucin compound is sufficient. Internally the mucin tabloids are administered to the number of one or two before and after meals." This employment of mucin as a remedial agent is attractive, but the probability of its specific value appears to us to be actually diminished by such a report on a case as is given at page 28. It deals with an old man of 96 who, while otherwise in perfect health, suffered from slight deafness and tinnitus. "I had no doubt that this was due to a senile change in the middle ear from desiccation of the mucous membrane. Such was proved to be the case by the success of the treatment—viz., injection of warm mucin solution up the Eustachian tubes—as his hearing improved at once and the tinnitus abated, and after a few injections disappeared." There is nothing in the facts cited to suggest that the cure would not have been equally effected by any application of moisture.

The book contains a few apparent contradictions which require explanation. Thus, much stress is laid upon the antiseptic qualities of mucus, and two series of experiments are quoted to establish the fact. That mucus possesses this quality we are prepared to admit, though its activity must be minimal, for bile, the most mucus-laden of all the body secretions, is very easily infected by micro-organisms. But assuming its antiseptic nature, the author leaves us with the following difficulty, that patients affected with hypertrophic rhinitis are the most inveterate subjects of the common cold, while the absence of liability to this infection is one of the cardinal characteristics of such hypomyxiatous people as the victims of rhinitis sicca. Again, on page 19 we are told that "there is but moderate provision for the production of mucus in the urinary organs," while later (in support of the thesis that where mucus is normally least abundant, there malignant disease is most common), the author speaks as follows: "the urinary tract, again, is wonderfully exempt from cancer, and here, too, mucus exists in considerable quantities on the surface lining."

Space does not permit more than a mention of the suggestion thrown out that carcinoma depends upon a thinning of the intercellular mucin, with the result that the body cells, being no longer properly bounded, are able to run riot and penetrate their surroundings; nor can we do justice to the imagination which suspects that sarcoma in the young is the expression of an inherited hypomyxia. It is clear that Mr. Stuart-Low is blessed with ideas, which is a hopeful condition in any man of science, but we cannot pretend to be sanguine of the fruits of this particular study.

[1] Mucous Membranes. Baillière, Tindall and Cox, 1905, 2s. 6d. net.

